# Criminal Mania

**Published:** 1896-04-18

**Authors:** F. M. F. Skene


### Criminal Mania.

By F. M. F. Skene.

### II.—THE PLEA OF INSANITY.

In our last issue, on the subject of criminal mania, we stated our conviction, founded on unusual opportunities of examining such cases, that there are certain symptoms of genuine mental disease by which it can generally be distinguished from the undoubted guilt that often personates it as a means of escape from the penalties of crime, and also from the discreet veil of supposed insanity, which is thrown over the criminality of persons of rank or good position as the only possible explanation of their apparent depravity. To take the first and most important of these indications, we believe it may be laid down as an unerring principle that where a crime is committed from which no possible advantage, real or fancied, can accrue to the perpetrator, even by the gratification of some evil impulse or momentary burst of passion, the case is one of true criminal mania, and the same may be assumed if the advantage to be derived from the crime is dependent on a complete hallucination; but where an offence is committed which has been prompted by the deliberate purpose and expectation of direct personal benefit—even though that benefit be only the satisfaction of utterly depraved instincts—it may be taken as certain that it is not an instance of criminal mania, but of simple inbred corruption. Again, when a person committing a crime is honestly convinced, by the delusion of a distorted moral sense, that the deed is in reality a virtuous action he may justly be sheltered under the ægis of criminal insanity; but where the offender is perfectly aware of the culpability of the act, and yet deliberately proceeds with it, we may feel certain that he has no excuse in mental aberration, however inconsistent with his social position or antecedents the crime may be. A few cases in point will best illustrate these principles, and circumstances have placed many such at our disposal; but before we enter on these details, we are anxious to show that the deep importance of the subject to members of the medical profession has far-reaching ramifications, of which the gravity and extent may not as yet perhaps have been fully estimated by those whom it most concerns. The responsibility which devolves on medical men to give the final decision whenever the supposed insanity of a criminal is brought forward as a plea for mercy in the courts of justice is, of course, perfectly well known; and in the case of persons accused under the capital charge it renders them simply the arbiters of life and death. A man is put on his trial for wilful murder; it is proved beyond question that he and none other committed the crime; there is but one possible means of escape for him from the extreme penalty of the law, and that is the plea of insanity, and his counsel puts it forward on his behalf; the evidence of aberration of mind which he is able to bring forward is not of sufficient weight, and even if it were, a medical pronouncement on the subject is absolutely necessary. In the case of lighter offences, involving only terms of imprisonment more or less long, the opinion of the medical officer of the prison in which the culprit is confined, as to his mental condition, would be held decisive, and he would be dealt with accordingly. But where the rejection or the acceptance of the plea of insanity is to result either in the man's execution, or his being sentenced to be "detained during Her Majesty's pleasure," a medical expert is generally appointed by the Home Secretary to visit the criminal, to examine him thoroughly, usually during a period of some days, and to adjudicate finally upon the question; so that it rests absolutely on his decision whether he consigns the man to the hangman's rope, or to a quiet country home at Broadmoor for the term of his natural life. A case of this kind is invariably a matter of public notoriety. The newspapers have carried full details of the matter into every household, and the widespread interest it excites far and wide, has an influence on the most serious danger which threatens our national well-being, in a future that is not perhaps so far distant as we could wish it to be. It is sufficiently well known that we have arrived at a period in this *fin de siècle* when our brothers of the proletarian classes are in the habit of criticising most keenly the authoritative enactments and social conditions of the Imperial *régime* under which they live. That such is the case as regards the government of the country—the laws affecting land and labour, and other vexed questions in the region of politics—is made clearly manifest from day to day in many ways; but it is less generally known that the administration of justice in what they hold to be its glaring inconsistency is the one subject which, above all others, rouses their passions and moves them in the direction of animosity to constitutional authority. This is a matter which, of course, touches most nearly those who have been included wholesale under the opprobrious term of the "criminal classes," and therefore the extent to which it affects them, does not attract more notice than the sentiments of that portion of the community on any subject whatever, would be likely to receive. Yet, if there comes a time when this country is convulsed by a struggle of class against class, the most perilous element in that social upheaval will be due to a deeply-rooted sense of injustice in the exercise of the power of the law, especially as regards the death penalty.

The cause of this dangerous inequality in the distribution of sentences is not far to seek—speaking generally, and apart from the question of medical responsibility in exceptional cases—inasmuch as it lies in the very constitution of human nature. Her Majesty's Judges, in common with all their fellow-creatures, vary greatly in disposition, and in their opinion of the crimes with which the prisoners brought before them are charged. We need scarcely say that we gladly admit the distinguishing characteristic of the whole of their illustrious body to be a most noble sense of duty, and an unfailing desire to act with perfect impartiality and righteousness in the discharge of their onerous functions; yet this common basis of their judicial actions cannot prevent them from taking such views of the cases submitted to them as accord with their individual temperament, and on this the fate of criminals depends. Trial by jury is supposed to leave the decision as to their guilt and its extent in the hands of the twelve men, good and true, who are taken from their ordinary avocations to hear the details of the crime and deliver their verdict; but, as a matter of fact, they naturally follow the direction of the judge in all cases which involve the possible infliction of the capital penalty. According as his charge indicates the guilt or innocence or insanity of the prisoner, their verdict is given. In so many words, the judge's opinion decides all questions of life or death, unless he has to transfer his responsibility to the medical expert.

We must defer to our next issue a further elucidation of this subject and its bearing on the medical questions with which we are chiefly concerned.

